# Corrosion Inhibition in CO_2_-Saturated Brine by Nd^3+^ Ions

**DOI:** 10.3390/molecules28186593

**Published:** 2023-09-13

**Authors:** Jorge Canto, Roberto Ademar Rodríguez-Díaz, Lorenzo Martinez Martinez-de-la-Escalera, Adrian Neri, Jesus Porcayo-Calderon

**Affiliations:** 1 Corrosion y Proteccion (CyP), Buffon 46, Mexico City 11590, Mexico; canto@corrosionyproteccion.com (J.C.); lmm@corrosionyproteccion.com (L.M.M.-d.-l.-E.); adrian.neri@corrosionyproteccion.com (A.N.); 2 Department of Materials Engineering, Technological of Superiors Studies of Coacalco, Av. 16 de Septiembre 54, Cabecera Municipal, Coacalco 55700, Mexico; ademar@tesco.edu.mx; 3 Department of Chemical Engineering and Metallurgy, University of Sonora, Hermosillo 83000, Mexico

**Keywords:** Nd^3+^ ions, corrosion, neodymium carbonate, corrosion inhibitor, electrochemical measurements

## Abstract

This study reports the use of an inorganic corrosion inhibitor to mitigate dissolved CO_2_-induced corrosion. Using electrochemical techniques (polarization curves, open circuit potential, polarization resistance, and electrochemical impedance), the effect of adding Nd^3+^ ions on the corrosion resistance of X52 steel immersed in CO_2_-saturated brine at 20 °C and 60 °C was evaluated. The polarization curves showed that the Icorr values tend to decrease with increasing Nd^3+^ ion concentration, up to the optimal inhibition concentration, and that the corrosion potential increases at nobler values. Open circuit potential measurements showed a large increase in potential values immediately after the addition of the Nd^3+^ ions. Similarly, polarization resistance measurements showed similar trends. It was observed that regardless of temperature, Nd^3+^ ions can reduce the corrosion rate by more than 97% at doses as low as 0.001 M. Electrochemical impedance measurements confirmed the formation of a protective layer on the steel surface, which caused an increase in the magnitude of the impedance module and phase angle, which indicates an increase in the resistance to charge transfer and capacitive properties of the metallic surface. The characterization of the metallic surface showed that the protective layer was formed by Nd carbonates, whose formation was due to a CO_2_ capture process.

## 1. Introduction

One of the main problems affecting the oil industry is the degradation of materials caused by the presence of dissolved CO_2_. Its impact on the reliability of the processes has motivated its constant study [1,2,3] and the way to avoid its destructive effect [4,5,6,7]. Since the construction materials used in the manufacture of pipelines for the transportation of hydrocarbons are Fe-based alloys, they are susceptible to corrosion in acidic environments such as those generated by the presence of dissolved CO_2_ [1,2,3,8,9].

For this reason, a common practice is the use of corrosion inhibitors that allow the retardation of the inevitable degradation process [4,5,6,7]. Its correct choice and application allow a significant reduction (at least 90%) of the steel corrosion rate. This is achieved by an adsorption process of the inhibitor molecules onto the metal surface by blocking the active sites and forming a physical barrier between the electrolyte and the metal surface. The most used corrosion inhibitors in the oil industry are organic compounds that have shown high efficiencies in inhibiting the corrosion process [4,5,6,7].

However, also inorganic compounds have proven to be efficient corrosion inhibitors, in particular, inhibitors based on rare earth elements have constantly attracted attention in environments rich in chlorides [10,11,12,13,14,15,16,17], but in the absence of CO_2_. In this sense, only a couple of studies have been reported on the use of compounds based on rare earths as corrosion inhibitors in CO_2_-saturated environments [18,19].

Although in both conditions the rare earths form protective layers, their protective mechanism is completely different. In CO_2_-free environments, rare earths precipitate as oxides or oxyhydroxides due to an increase in pH at the cathode sites caused by the O_2_ reduction reaction [11,12,14,20,21,22]; however, in the presence of dissolved CO_2_, they precipitate as rare earth carbonates due to a CO_2_ capture mechanism [19].

The most abundant rare earths are La, Ce, Nd, and Pr, and their toxicity in the form of chlorides is similar to that of NaCl [14,22,23]; therefore, their use as corrosion inhibitors can be considered safe for replacing inhibitors based on chromates (CrO^2−^_4_), mercurates, or others, which are highly toxic [20].

The studies reported in CO_2_-rich environments have used compounds based on Pr and La [18,19], therefore, in this study the corrosion inhibitor capacity of Nd^3+^ ions (such as NdCl_3_) is explored. Its performance is evaluated on an X52 steel in a Cl-rich solution (NaCl, 3.5% by weight) under dissolved CO_2_ saturation conditions at different temperatures (20 °C, 60 °C). Its evaluation is carried out through potentiodynamic polarization tests, open circuit potential, polarization resistance, and electrochemical impedance.

## 2. Results and Discussion

### 2.1. Potentiodynamic Polarization Curves

**Figure 1:**
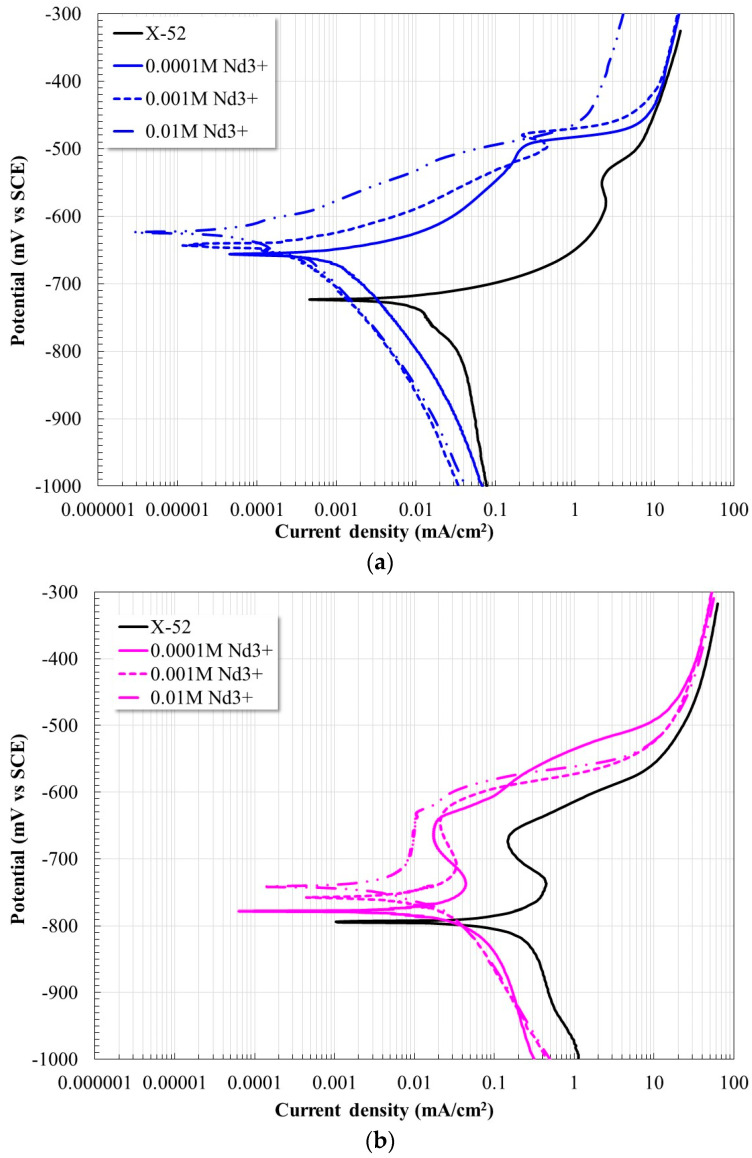
Polarization curves of X52 steel in CO_2_ saturated brine at different concentrations of Nd^3+^ ions after 24 h of immersion; (**a**) 20 °C, (**b**) 60 °C.

Figure 1 shows the potentiodynamic polarization curves of the X52 steel at 20 °C and 60 °C with and without the addition of Nd^3+^ ions at different concentrations. In the absence of an inhibitor, X52 steel showed an increase in its active behavior with increasing temperature. At 20 °C, the anodic branch shows an active behavior above its corrosion potential; at higher temperatures, the active zone is reduced possibly due to the precipitation of corrosion products due to a higher dissolution rate [3,8,24]. This is evident by looking at the cathode branch, which shifts to higher current densities with increasing temperature. In the presence of the inhibitor, in all cases, a displacement of the polarization curves was observed toward lower corrosion densities and toward more noble potentials. This shift was greater with increasing inhibitor concentration. The anodic branches show a decrease in the active behavior of the steel, and with the increase in the concentration of the inhibitor, they develop a pseudo-passive zone. Similarly, the cathode branches show a shift at lower current densities.

It is evident that the presence of Nd^3+^ ions caused the observed changes, a decrease in the rate of both the anodic and cathodic processes [18,19]. Consistent with the changes observed in the anodic branch, this was due to the formation of a carbonate-based protective film as already demonstrated in a previous study [19].

**Figure 2:**
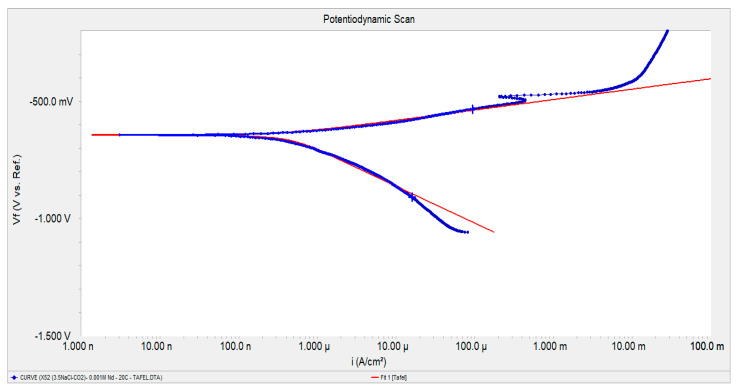
Fitting of the polarization curves through the software Gamry Echem Analyst (version 6.03).

From the polarization curves, the electrochemical parameters shown in Table 1 and Table 2 were obtained. The fitting of the polarization curves was performed on the Tafel regions using the Gamry Echem Analyst software (version 6.03) as illustrated in Figure 2.

**Figure 3:**
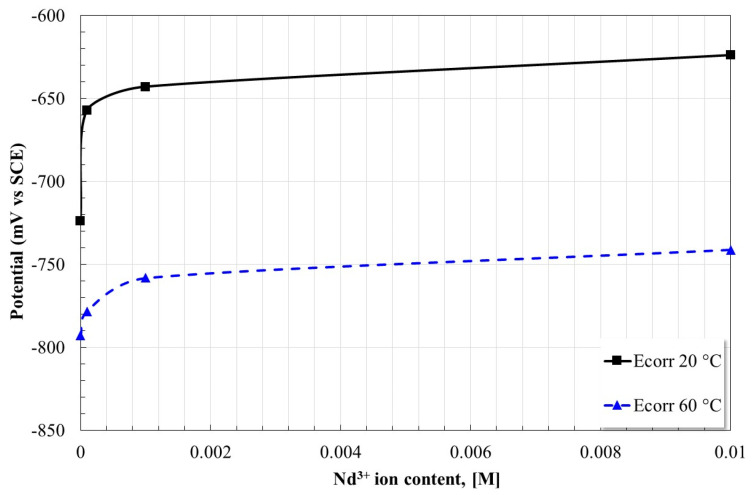
Effect of Nd^3+^ ion concentration on the corrosion potential of X52 steel in CO_2_ saturated brine at different temperatures.

Based on the tabulated data, Figure 3 shows the effect of the inhibitor concentration on the Ecorr of the X52 steel. As already indicated, the increase in temperature caused a more active behavior, but on the other hand, the increase in inhibitor concentration caused a more noble behavior, this is associated with the formation of a protective layer on the steel surface [19].

**Figure 4:**
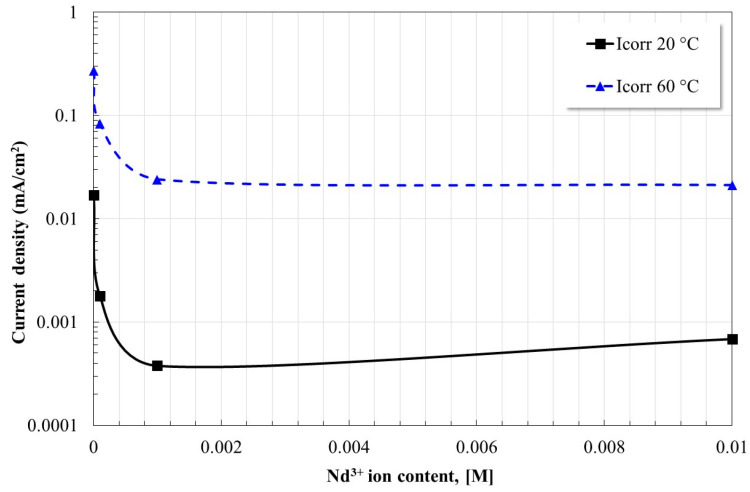
Effect of Nd^3+^ ion concentration on the corrosion current density of X52 steel in CO_2_ saturated brine at different temperatures.

Figure 4 shows the effect of inhibitor concentration on the corrosion current density (Icorr) of X52 steel. In general, it is observed that the decrease in the Icorr values is greater with the increase in the concentration of the inhibitor. At 20 °C the decrease is up to two orders of magnitude, and at 60 °C up to one order of magnitude. Once again, the presence of Nd^3+^ ions has an inhibitory effect on corrosion due to the formation of a protective layer based on carbonates [19]. As it has already been reported, the reduction in Icorr values due to the presence of lanthanide ions in CO_2_-saturated environments is greater because the compounds that form the protective layers are different from those formed in aerated systems [18,19], as will be noted in the analysis of corrosion products in Section 2.5. Studies reported at 25 °C indicate Icorr values between 0.004–0.016 mA/cm^2^ depending on the concentration of inhibitor (Pr(4OHCin)_3_) and chloride ions [18], on the other hand, in more recent studies and conditions similar to those reported here, Icorr values of 0.013 mA/cm^2^ were indicated for the optimal concentration of inhibition (1 mM LaCl_3_) at 60 °C [19]. Values were very similar, in both cases, to those obtained in this study.

The inhibition efficiencies calculated show that Nd^3+^ ions reduce the corrosion rate of X52 steel significantly in the concentrations evaluated and that the increase in temperature reduces their effectiveness in an insignificant way at concentrations above 0.1 mM.

### 2.2. Open Circuit Potential Measurements

**Figure 5:**
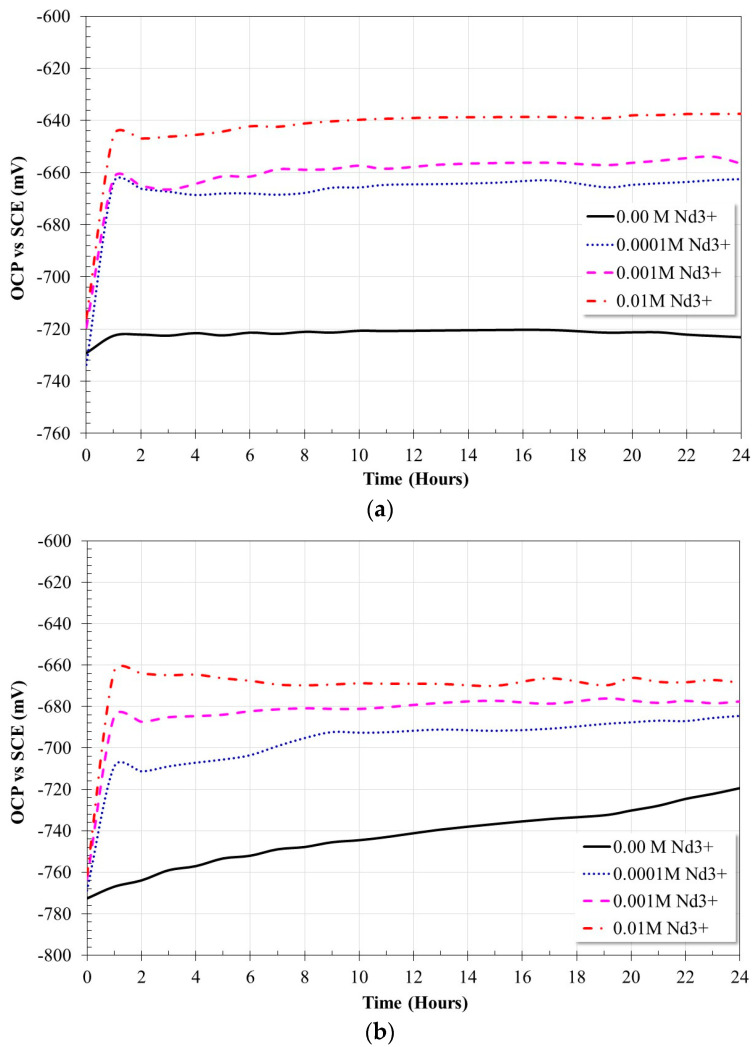
Evolution of OCP values for X52 steel in CO_2_ saturated brine at different concentrations of Nd^3+^ ions; (**a**) 20 °C, (**b**) 60 °C.

Figure 5 shows the variation in OCP values for X52 steel in a 3.5% NaCl solution saturated with CO_2_ with different inhibitor additions at different temperatures.

In the absence of an inhibitor, at the beginning of the test, the X52 steel showed a more active behavior with the increase in temperature. At 20 °C, the OCP values remain oscillating around −720 mV; however, at 60 °C, the OCP values showed a constant increase. These trends suggest that, at 20 °C, the electrochemical activity of the steel surface was quasi-stable; however, at 60 °C, it presented higher activity with a tendency to reduce its active state because of the formation or accumulation of corrosion products on its surface.

In the presence of an inhibitor, once added, and regardless of the test temperature, all measurements showed the same behavior, namely a sudden increase followed by a decrease. It has been reported that the abrupt changes at the beginning of the test may be due to a rearrangement of surface layers because of an increase in anodic activity or a reduction in the cathodic process. [14,15,20]. However, the subsequent decrease in previous studies was shown to be due to a sudden reduction in dissolved CO_2_ concentration due to a CO_2_-capture process in the presence of lanthanide ions [19]. After this perturbation, it was observed that the OCP values tend to be noble values when increasing the concentration of inhibitor. In general, and regardless of the test temperature, it is observed that the displacement in the OCP values does not exceed 85 mV. This indicates that Nd^3+^ ions act as a mixed inhibitor [19,25,26,27].

### 2.3. Linear Polarization Resistance Measurements

**Figure 6:**
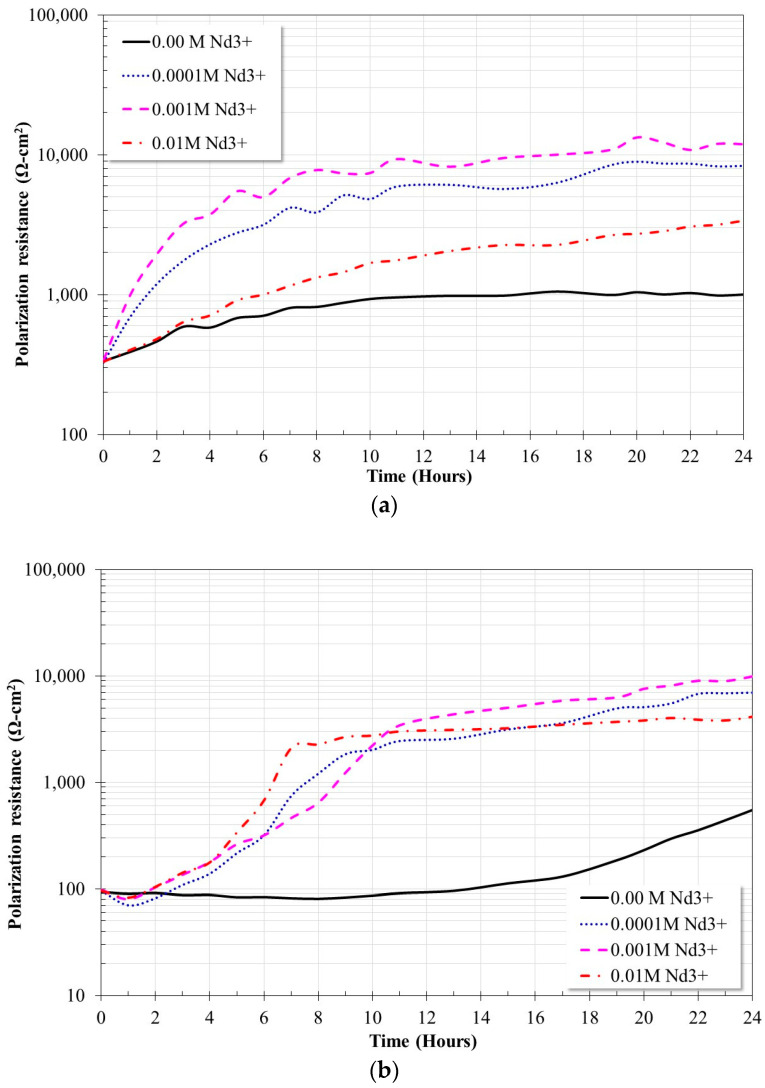
Evolution of polarization resistance values for X52 steel in CO_2_ saturated brine at different concentrations of Nd^3+^ ions; (**a**) 20 °C, (**b**) 60 °C.

Figure 6 shows the evolution of the Rp values for the X52 steel immersed in a 3.5% NaCl solution saturated with CO_2_ at 20 °C and 60 °C, with and without the addition of a corrosion inhibitor. In the absence of an inhibitor, X52 steel shows a decrease in its Rp values with increasing temperature. The observed trend is consistent with the evolution of their OCP values. At 20 °C it shows a constant increase from 300 to 1000 Ω-cm^2^ in the first 10 h of immersion and remains constant for the rest of the test. At 60 °C, its Rp values oscillate around 90 Ω-cm^2^ in the first 10 h, and a constant increase is subsequently observed for the rest of the test.

With the addition of the inhibitor, the evolution of the Rp values was dependent on the temperature and concentration of Nd^3+^ ions. At 20 °C, the Rp values tended to increase with immersion time. The maximum values of Rp were obtained with the addition of 0.001 M of inhibitor, at higher concentrations their values were lower. This has been associated with the increase in the concentration of Cl^-^ ions by the addition of NdCl_3_ [19]. At 60 °C, at the beginning of the test, a slight decrease in the Rp values was also observed, followed by a constant increase until the end of the test. The maximum Rp values were obtained with an inhibitor concentration of 0.001 M.

**Figure 7:**
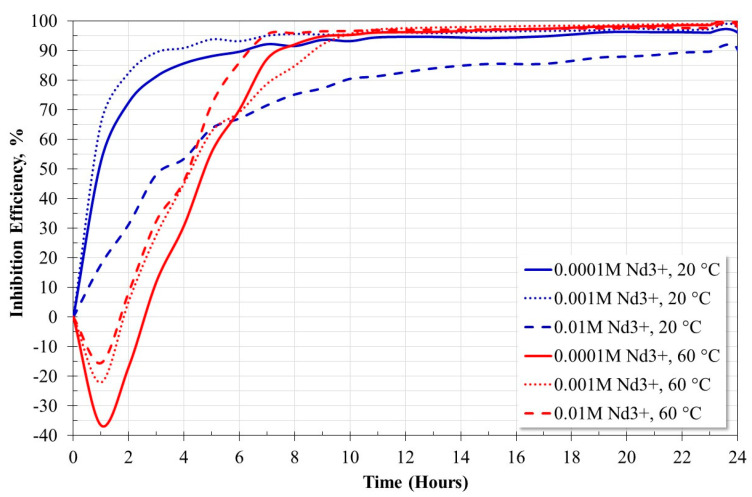
Effect of Nd^3+^ ion concentration and temperature on inhibition efficiency.

Based on the Rp values (Figure 6), the inhibition efficiency of the Nd^3+^ ions was determined (Figure 7). At 20 °C, the inhibition efficiency increased steadily reaching values of 97% for an inhibitor concentration of 0.001 M. At 60 °C, the inhibition efficiency apparently decreased after the inhibitor was added. However, as established in previous work [19], this was due to a rapid capture reaction of dissolved CO_2_, and a subsequent restoration of the saturation concentration of CO_2_. After this event, the inhibition efficiency tended to increase constantly until the end of the trial. At 60 °C, inhibition efficiencies greater than 97% were obtained at all the concentrations evaluated, in addition, they were achieved in the shortest time compared to what was observed at 20 °C. The maximum inhibition efficiency was 99% for an inhibitor concentration of 0.001 M. The inhibition efficiencies obtained show that rare earths are more efficient corrosion inhibitors in CO_2_-saturated saline solutions due to the formation of protective layers based on carbonates [19] unlike the inhibition efficiencies reported in aerated saline solutions whose protective layers are based on oxides or hydroxides [15,16,17]. Table 3 shows the average of the values of polarization resistance and the inhibition efficiency of the last four hours of the corrosion test.

### 2.4. Electrochemical Impedance Spectroscopy Measurements

**Figure 8:**
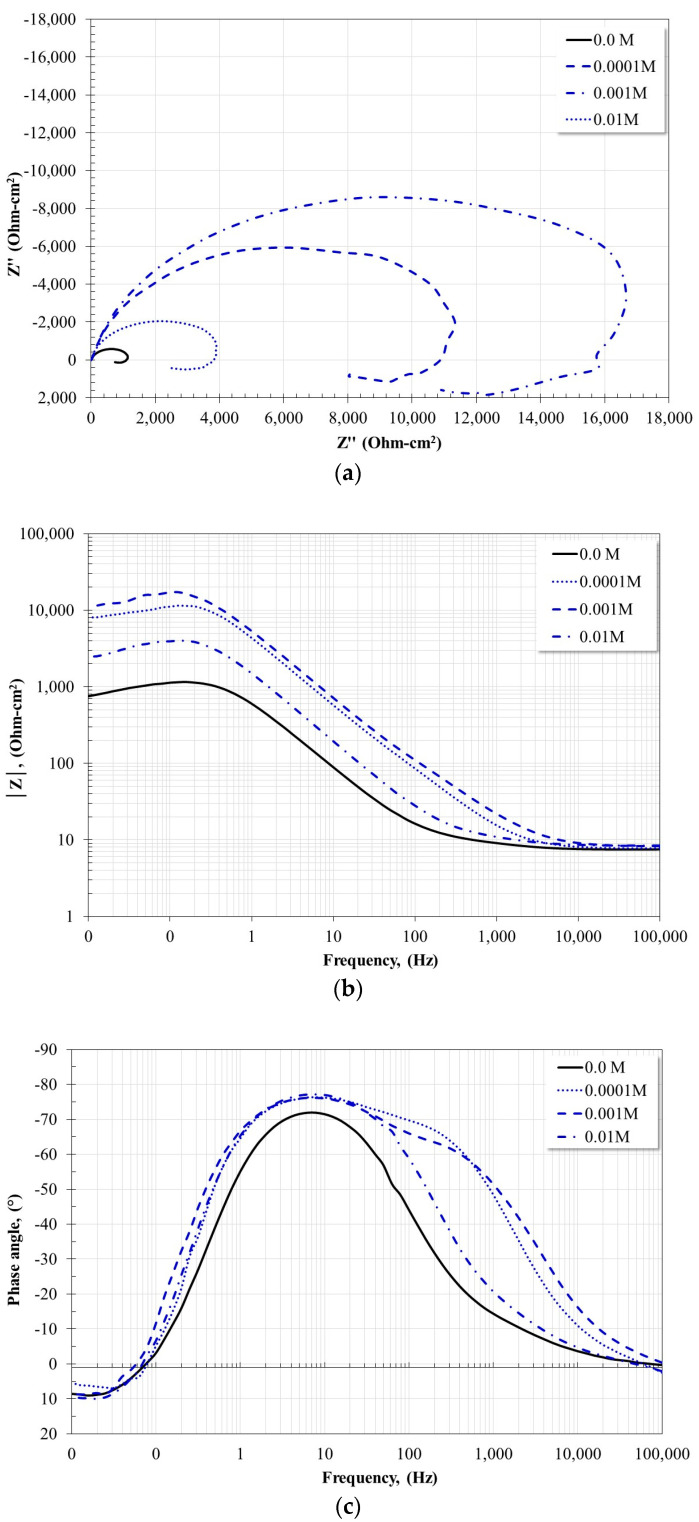
Nyquist and Bode diagrams for X52 steel after 24 h of immersion in CO_2_-saline solution at 20 °C and different inhibitor concentrations. (**a**) Nyquist diagram; (**b**) Bode plot-impedance modulus; (**c**) Bode plot-phase angle.

**Figure 9:**
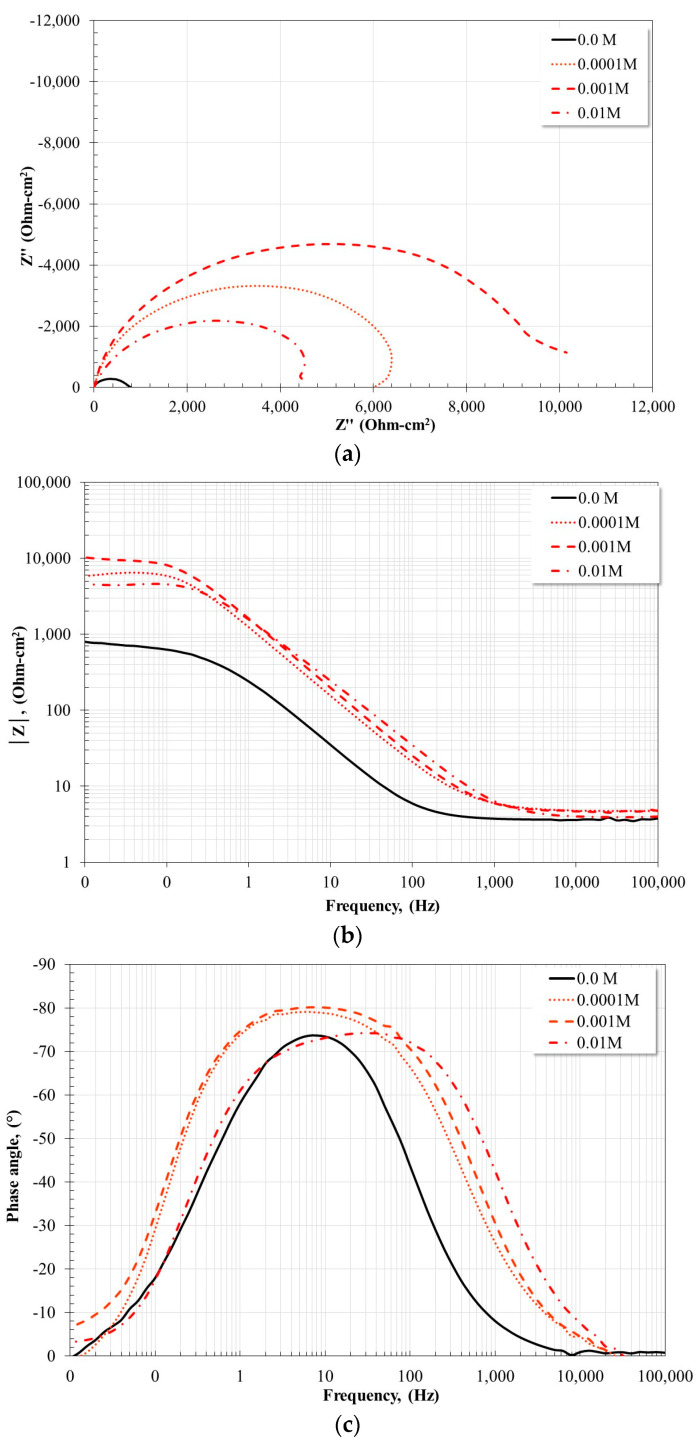
Nyquist and Bode diagrams for X52 steel after 24 h of immersion in CO_2_-saline solution at 60 °C and different inhibitor concentrations. (**a**) Nyquist diagram; (**b**) Bode plot-impedance modulus; (**c**) Bode plot-phase angle.

Figure 8 and Figure 9 show the Nyquist and Bode plots for the X52 steel after 24 h of immersion in a CO_2_-saturated saline solution at 20 °C and 60 °C with and without the addition of corrosion inhibitor. At 20 °C (Figure 8), the electrochemical impedance spectra obtained after 24 h of immersion show similar characteristics. In all cases, the Nyquist diagram shows the apparent presence of a single capacitive semicircle and the development of an inductive loop. The smallest diameter of the capacitive semicircle was obtained in the absence of the inhibitor, and the largest diameter with the addition of 0.001 M inhibitor. The Bode diagram in its impedance modulus format, |Z|, shows characteristics consistent with what is observed in the Nyquist diagram, namely, in the absence of an inhibitor, the formation of a high-frequency plateau is observed starting at 1000 Hz, and in the presence of the inhibitor its development occurs at higher frequencies. At intermediate frequencies, the apparent formation of a single linear relationship, log f-log |Z|, is observed, and at low frequencies, the impedance modulus tends to decrease due to the formation of the inductive loop observed in the Nyquist diagram. However, the Bode plot in its phase angle format indicates that in the absence of an inhibitor only one time constant is detected, with a phase angle maximum of −72° around 6 Hz. This corresponds to a single capacitive semicircle and a single linear relationship, log f-log |Z|. However, in the presence of the inhibitor, the presence of two time constants is observed. The first time constant is around 60 Hz with phase angle maxima between 60–65° for inhibitor concentrations of 0.0001 M and 0.001 M, and the second time constant is located at 6 Hz and the phase angle maximum phase is about 78° for all concentrations. These characteristics suggest that, in the presence of the inhibitor, a protective layer with porous characteristics was formed on the steel surface.

At 60 °C (Figure 9), the Nyquist diagram also shows the apparent presence of a single capacitive semicircle with the incipient presence of a small inductive loop in the low-frequency region. In the absence of the inhibitor, the smallest diameter of the capacitive semicircle was obtained, and with the addition of 0.001 M the largest diameter was obtained. The Bode plot in its impedance modulus format, |Z|, in the high-frequency region, the formation of the high-frequency plateau is observed. In the absence of the inhibitor, it begins to form at frequencies below 1000 Hz and, in the presence of the inhibitor, at frequencies slightly higher than 1000 Hz. At intermediate frequencies the apparent formation of a single linear relationship is observed, log f-log |Z|, and at low frequencies, the formation of the low-frequency plateau is defined. The Bode plot in its phase angle format, in the absence of the inhibitor, shows the presence of a time constant, with a phase angle maximum of −72° around 8 Hz, similar to that observed at 20 °C. In the presence of the inhibitor, the apparent presence of a time constant with a very large maximum is observed, this suggests that it is two overlapping time constants. The largest phase angle occurs for the inhibitor concentration of 0.001 M, being −80° around 8 Hz. This suggests the formation of a dense and compact protective layer, as has been shown in a previous study [19].

The inductive loop observed in all cases has been attributed to relaxation processes associated with the adsorption of some intermediate species formed during the anodic dissolution process [28,29,30,31,32] or the redissolution of the passive surface [31,32]. In the absence of an inhibitor, it has been associated with the adsorption of species such as FeOH_ads_ [3] and, in the presence of the inhibitor, it may be due to the adsorption of carbonate compounds. On the other hand, the magnitude of the phase angle provides an idea of the corrosion resistance of the system, namely, an increase in the phase angle implies a more capacitive behavior of the electrochemical interface [31].

**Figure 10:**
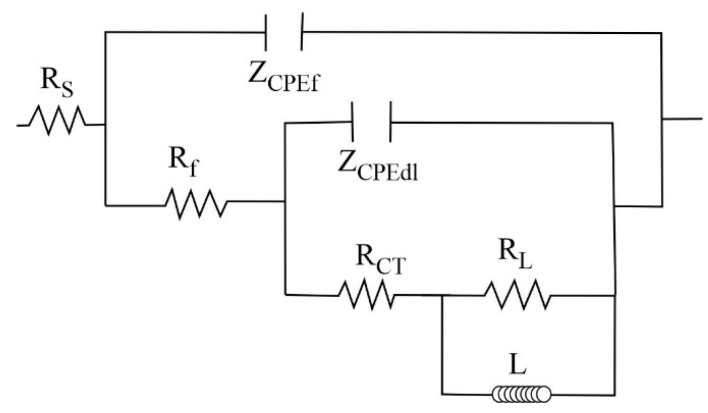
Equivalent circuit used to fit the impedance spectra.

Based on what was previously discussed, the impedance spectra were adjusted to the equivalent circuit shown in Figure 10. Instead of the capacitance (C), the constant phase element (*CPE*) has been used, whose impedance has been defined according to:

(1) $$Z_{CPE}=\left(\frac{1}{Y_{0}}\right){\left(j\omega \right)}^{-n},$$

In this expression, *Y*_0_ is the magnitude of the *CPE*, *j* is √-1, *ω* is the angular frequency, and *n* is a deviation parameter associated with the heterogeneities of the working electrode surface. Depending on the value of *n*, the behavior of the *CPE* has been associated with an inductor (*n* = −1), a resistor (*n* = 0), Warburg impedance (*n* = 0.5), or a capacitor (*n* = 1).

In the equivalent circuit, the time constant Z_CPEdl_-R_CT_ represents the capacitive-resistive response of the metallic surface, where Z_CPEdl_ is the impedance of the double layer and R_CT_ is the resistance to charge transfer, the time constant Z_CPEf_-R_f_ represents the capacitive-resistive response of the carbonate layer, where Z_CPEf_ is its impedance and R_f_ its resistance, and the R_L_-L time constant represents the resistance associated with the adsorption process of species and the inductive process observed in the impedance spectra, and Rs the resistance of the solution (between the surface of WE and CE).

Table 4 and Table 5 show the adjustment parameters obtained (χ^2^ < 0.001). Since the Rp values can be represented as the sum of all the resistances involved in the impedance measurements (Rp ≈ R_f_ + R_CT_ + R_L_), the ΣR column shows this result. The comparison of these results with the last value of Rp reported in Figure 6 shows that they are similar. This suggests that the proposed equivalent circuits adequately represent the surface processes observed in the impedance spectra. Studies reported at 25 °C indicate R_CT_ values between 3000–11,000 Ω-cm^2^ depending on the inhibitor concentration (Pr(4OHCin)_3_) and chloride concentration [18], and under conditions similar to those reported here at 60 °C impedance modulus values (|Z| ≈ R_CT_) of the order of 5000 Ω-cm^2^ are reported for the optimal concentration of inhibition (1 mM LaCl_3_) [19]. Values similar to those reported here without considering the substrate.

### 2.5. X-ray Diffraction Analysis

**Figure 11:**
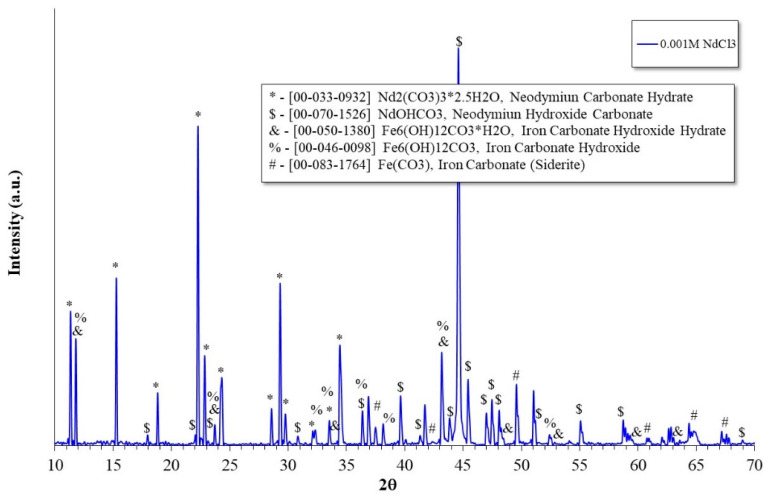
X-ray diffraction pattern of the corrosion products of steel X52 evaluated at 60 °C with the addition of 0.001 M NdCl_3_.

X-ray diffraction analysis on the surface of the samples confirmed the majority presence of Nd-carbonates, and minor amounts of Fe-carbonates, as shown in Figure 11. Similar spectra were obtained with the other test conditions in the presence of the inhibitor.

This confirms that the Nd^3+^ ions precipitate as a protective layer of carbonates, thereby reducing the corrosion rate of the X52 steel. Unlike CO_2_-free systems where rare earth ions form protective layers based on oxides and/or hydroxides [12,15,16,17,33,34,35,36], this study corroborates that in systems with dissolved CO_2_, lanthanide ions react through a CO_2_-capture mechanism, favoring the precipitation of rare earth carbonates [19]. It has been reported that due to their free energy of formation, rare earth carbonates [Ln_2_(CO_3_)_3_] are thermodynamically the most stable species compared to their respective oxides and hydroxides [37], and their precipitation occurs according to the following reaction overall [19,37]:

(2) $$2Ln_{\left(aq\right)}^{3+}+3CO_{3_{\left(aq\right)}}^{2-}\overset{}{↔}Ln_{2}{\left(CO_{3}\right)}_{3_{\left(s\right)}},$$

This difference with respect to CO_2_-free systems is due to the fact that during the CO_2_ dissolution process, hydrated CO_2_ produces hydrogen ions and carbonate ions [3]. This causes the main cathodic reaction to be the hydrogen evolution reaction, and the carbonate ions react with the lanthanide ions according to the previous reaction. It has been suggested that the dissociation reaction of dissolved CO_2_ (H_2_CO_3_) occurs at the steel-electrolyte interface [9]; therefore, the availability of the carbonate ion is greater, favoring its precipitation as a protective layer on the steel surface.

## 3. Materials and Methods

Metallic samples were used, obtained from an API X52 steel duct, with dimensions of 10 × 10 × 5 mm. The electrical connection of the samples was made with a Cu wire welded using the spot-welding technique. In this condition, the samples were encapsulated in epoxy resin. For the corrosion tests, the encapsulated samples were roughened with silicon carbide paper up to grade 600, washed with distilled water and ethanol, and dried with hot air.

The corrosive medium was a solution of NaCl (3.5% by weight) saturated with CO_2_, and the test temperatures were 20 °C and 60 °C. A constant bubbling of CO_2_ was maintained for at least one hour prior to the start of the tests, and this prevailed until the end of the tests. The corrosion inhibitor used was neodymium chloride (NdCl_3_) at concentrations of 0.0001 M, 0.001 M, and 0.01 M. For simplicity, the concentration of the inhibitor will be referred to as the concentration of Nd^3+^ ions, that is, 0.0001 M Nd^3+^, 0.001 M Nd^3+^, and 0.01 M Nd^3+^.

An electrochemical cell with three electrodes was used, where the working electrodes were the encapsulated samples of X52 steel, the reference electrode encapsulated saturated calomel (SCE), and as an auxiliary electrode, a graphite rod with an area greater than that of the working electrode was used. Potentiodynamic polarization curves, open circuit potential (OCP), linear polarization resistance (RPL), and electrochemical impedance spectroscopy (EIS) measurements were performed. Prior to any measurement, the working electrode was immersed in the electrolyte 15 min prior to achieving a steady state. Electrochemical measurements were performed for 24 h with a GAMRY potentiostat/galvanostat (model 1100).

The potentiodynamic polarization curves were performed after 24 h of immersion to allow the formation of a protective layer of carbonates. For this, the working electrode was polarized from −300 mV to 1000 mV, with respect to the corrosion potential (Ecorr), at a scan rate of 1 mV/s. OCP and LPR measurements were performed at one-hour intervals. For LPR measurements, the working electrode was polarized ±10 mV from its open circuit potential at a sweep rate of 10 mV/min. The EIS measurements were made by applying to the working electrode an alternating current signal with an amplitude of 10 mV in a frequency range of 100 kHz to 0.01 Hz. In these cases (OCP, LPR, EIS), the addition of the inhibitor to the corrosive medium was performed after the first measurement (zero time). The inhibition efficiency was determined according to the following equation:

(3) $$E\left(\%\right)=\left(\frac{Rp_{i}-Rp_{b}}{Rp_{i}}\right)*100,$$

Therein, *Rp_i_* = polarization resistance in the presence of inhibitor, *Rp_b_* = polarization resistance in the absence of inhibitor.

## 4. Conclusions

Nd^3+^ ions added to saline solutions saturated with CO_2_ act as efficient corrosion inhibitors for X52 steel. Based on the electrochemical analysis carried out, it was found that they precipitate on the steel surface, forming a Nd-carbonate protective layer. Regardless of the temperature, its presence reduces both the anodic and cathodic processes, which causes a reduction in the corrosion rate and a nobler behavior. The reduction in active behavior caused an increase in Rp values and inhibition efficiencies. The inhibition efficiencies were higher than 97% at an optimal concentration of 0.001 M. In addition, the precipitation of the Nd carbonate layer causes an increase in the capacitive properties of the steel surface, due to its greater stability with respect to their respective oxides and hydroxides. It was confirmed that the precipitation mechanism of Nd^3+^ ions is due to a CO_2_-capture process.

## Figures and Tables

**Table 1:** Electrochemical parameters obtained from the Tafel regions at 20 °C.

[Nd^3+^] Molar Concentration	Ecorr [mV]	βa [mV/Dec]	βc [mV/Dec]	Icorr [µA/cm^2^]	Inhibition Efficiency, [%]
0.0	−724	35	347	17	---
0.0001	−657	57	201	1.78	89.5
0.001	−643	44	162	0.379	97.8
0.01	−624	55	122	0.688	95.9

**Table 2:** Electrochemical parameters obtained from the Tafel regions at 60 °C.

[Nd^3+^] Molar Concentration	Ecorr [mV]	βa [mV/Dec]	βc [mV/Dec]	Icorr [µA/cm^2^]	Inhibition Efficiency, [%]
0.0	−793	46	421	270	---
0.0001	−778	39	397	83	69.3
0.001	−758	38	183	24	91.1
0.01	−741	34	196	21	92.2

**Table 3:** Average of the Rp values and the inhibition efficiency of the last four hours of the corrosion test.

NdCl_3_ (M)	20 °C Rp (Ω·cm^2^)	20 °C Inhibition Efficiency (%)	60 °C Rp (Ω·cm^2^)	60 °C Inhibition Efficiency (%)
0.0001	8549.80	96.16	372.60	98.45
0.001	11,994.00	97.13	6255.60	98.86
0.01	3044.00	89.06	3926.20	97.56

**Table 4:** Electrochemical parameters of the impedance spectra after 24 h of immersion at 20 °C.

NdCl_3_ (M)	R_f_ (Ω·cm^2^)	Y_0f_ (Ω^−1^·cm^−2^·s^n^)	n	R_CT_ (Ω·cm^2^)	Y_0dl_ (Ω^−1^·cm^−2^·s^n^)	n_dl_	R_L_ (Ω·cm^2^)	L (H·cm^2^)	ΣR (Ω·cm^2^)
0	6.4	9.1836 × 10^−5^	0.92	770.3	1.7251 × 10^−4^	0.90	478	1614	1254.7
0.0001	463	3.3588 × 10^−5^	0.89	7753	6.8142 × 10^−6^	0.97	5728	10,079	13,944
0.001	261	2.6998 × 10^−5^	0.87	10977	7.9978 × 10^−6^	0.95	9127	23638	20,365
0.01	9.7	4.7457 × 10^−5^	0.94	2492	6.6498 × 10^−6^	0.90	2003	6126	4504.7

**Table 5:** Electrochemical parameters of the impedance spectra after 24 h of immersion at 60 °C.

NdCl_3_ (M)	R_f_ (Ω·cm^2^)	Y_0f_ (Ω^−1^·cm^−2^·s^n^)	n	R_CT_ (Ω·cm^2^)	Y_odl_ (Ω^−1^·cm^−2^·s^n^)	n_dl_	R_L_ (Ω·cm^2^)	L (H·cm^2^)	ΣR (Ω·cm^2^)
0	0.1	43364 × 10^−4^	0.78	664.1	3.326 × 10^−4^	0.97	68.41	3200	732.61
0.0001	18.42	1.0078 × 10^−4^	0.93	5966	4.4948 × 10^−5^	0.88	1616	6047	7600.42
0.001	15.41	4.872 × 10^−5^	0.99	10188	7.1392 × 10^−5^	0.82	3390	2313	13,593.41
0.01	63.91	4.7547 × 10^−5^	0.96	4664	1.2279 × 10^−4^	0.84	1058	172.06	5785.91

## Data Availability

Not applicable.
